# Optimization of Polystyrene Biodegradation by *Bacillus cereus* and *Pseudomonas alcaligenes* Using Full Factorial Design

**DOI:** 10.3390/polym14204299

**Published:** 2022-10-13

**Authors:** Martina Miloloža, Šime Ukić, Matija Cvetnić, Tomislav Bolanča, Dajana Kučić Grgić

**Affiliations:** Faculty of Chemical Engineering and Technology, University of Zagreb, Trg Marka Marulića 19, 10 000 Zagreb, Croatia

**Keywords:** microplastics, polystyrene, *Bacillus cereus*, *Pseudomonas alcaligenes*, Taguchi design, biodegradation, full factorial design

## Abstract

Microplastics (MP) are a global environmental problem because they persist in the environment for long periods of time and negatively impact aquatic organisms. Possible solutions for removing MP from the environment include biological processes such as bioremediation, which uses microorganisms to remove contaminants. This study investigated the biodegradation of polystyrene (PS) by two bacteria, *Bacillus cereus* and *Pseudomonas alcaligenes*, isolated from environmental samples in which MPs particles were present. First, determining significant factors affecting the biodegradation of MP-PS was conducted using the Taguchi design. Then, according to preliminary experiments, the optimal conditions for biodegradation were determined by a full factorial design (main experiments). The RSM methodology was applied, and statistical analysis of the obtained models was performed to analyze the influence of the studied factors. The most important factors for MP-PS biodegradation by *Bacillus cereus* were agitation speed, concentration, and size of PS, while agitation speed, size of PS, and optical density influenced the process by *Pseudomonas alcaligenes*. However, the optimal conditions for biodegradation of MP-PS by *Bacillus cereus* were achieved at *γ*_MP_ = 66.20, MP size = 413.29, and agitation speed = 100.45. The best conditions for MP-PS biodegradation by *Pseudomonas alcaligenes* were 161.08, 334.73, and 0.35, as agitation speed, MP size, and OD, respectively. In order to get a better insight into the process, the following analyzes were carried out. Changes in CFU, TOC, and TIC concentrations were observed during the biodegradation process. The increase in TOC values was explained by the detection of released additives from PS particles by LC-MS analysis. At the end of the process, the toxicity of the filtrate was determined, and the surface area of the particles was characterized by FTIR-ATR spectroscopy. Ecotoxicity results showed that the filtrate was toxic, indicating the presence of decomposition by-products. In both FTIR spectra, a characteristic weak peak at 1715 cm^−1^ was detected, indicating the formation of carbonyl groups (−C=O), confirming that a biodegradation process had taken place.

## 1. Introduction

It can be said that we live in the plastic age due to the extensive consumption of plastic products in everyday life. Global plastic production has been increasing since the 1950s, reaching 367 million tons in 2020 [1]. The most frequently produced are polyethylene (PE), polyvinyl chloride (PVC), polypropylene (PP), polystyrene (PS), and polyethylene terephthalate (PET) [2,3]. PS is widely used due to excellent properties such as good mechanical properties, lightweight, versatility, durability, stability, and low cost [4]. It is applicable for food and laboratory containers, disposable plastic accessories, CD/cassette boxes, toys, the automobile industry, and construction materials [5,6]. PS production reached 10.4 million tons in 2018, and it is estimated that global PS production will reach 10.9 million tons in 2024 [7]. During the manufacturing process, various additives such as UV stabilizers, antioxidants, flame retardants, and/or lubricants [4,8] are usually added to PS to improve the properties and applicability of the polymer [9]. Generally, stabilizers and antioxidants are added in an amount range between 0.05 and 3% (*w*/*w*), depending on the structure of the additive and the polymer. For example, phenolic antioxidants are added in small amounts in high-density polyethylene (HDPE), while phosphites are used in large amounts in high-impact PS (HIPS). In addition, dyes (e.g., azo dyes) are commonly used in PS to provide a bright, transparent color [9], but these compounds have a high migration potential. In addition, carcinogenic polybrominated diphenyl ethers [10,11], styrene oligomers as unintentional additives in expanded polystyrene (EPS) [12], and styrene monomers [13] can also migrate from PS products. This makes the leaching of additives from polymers a potential environmental risk to aquatic organisms. Accordingly, it is not surprising that accumulated MP-PS in the environment lead to changes in the ecosystem. Natural degradation of synthetic thermoplastic PS is a very slow process, but microorganisms can use it as a carbon source [4]. According to Auta et al. [14], PS pollutants persist under technical conditions (biodegradation by bacterial genera of *Bacillus*) for 363.16 (*Bacillus cereus*) and 460.00 days (*Bacillus gottheilii*). The environmental lifespan of PS ranges from 50 to 80 years [15]. Kim et al. [16] and Zhang et al. [17] reported that biodegradation of PS in natural ecosystems is slow and requires several hundred years for complete degradation. However, insect-based systems suggest that a much shorter period of biodegradation of PS can occur within a few weeks.

Bioremediation, as an economical, efficient, and environmentally friendly biological process [18], involves the use of microorganisms, bacteria, mold, and yeast [19] for the purpose of removing pollutants from the environment. Biodegradation processes influence many factors such as abiotic (pH-value, concentration of dissolved oxygen, moisture content, temperature, salinity, and presence or absence of UV radiation), biotic factors (type and number of microorganisms, extracellular enzymes, and biosurfactants), and properties of MP particles (structure, molecular weight, hydrophobicity, functional groups, etc.) [20]. Biodegradation of PS is complex, and it is considered that biodegradation begins after microorganisms colonize PS particles. Thereafter, PS may be degraded into smaller fragments such as oligomers, dimers, and/or monomers. Assimilation can occur due to the ability of microorganisms to use styrene as a carbon source [21]. Pathways for the biodegradation of PS by various enzymes (hydroquinone [22], oxidoreductases, laccases, lipases, P450 monooxygenases, and alkane hydroxylases [23]) have been proposed, which include conversion of the polymer to carboxylic acids and their further metabolism by *β*-oxidation and Krebs cycle [24]. The first proposed pathway is that styrene is converted into styrene epoxide by styrene monooxygenase and further into 4-maleylacetoacetate by styrene oxide isomerase, phenylacetaldehyde dehydrogenase, phenylacetate hydroxylase, 2-hydroxyphenylacetate hydroxylase, and homogentisate 1,2-dioxygenase. Through the *β*-oxidation pathway, 4-maleylacetoacetate is converted to acetyl-CoA, followed by citric acid (TCA) cycle to the central biosynthesis pathways. On the other hand, in the second proposed pathway of biodegradation of PS, styrene is hydroxylated by styrene dioxygenase on the aromatic ring to generate styrene cis-glycol, which can be further converted to acetyl-CoA by cis-glycol dehydrogenase, catechol 2,3-dioxygenase, 2-hydroxymuconic acid semialdehyde hydrolase, 2-hydroxypenta-2,4-dienoate hydratase, 4-hydroxy-2-oxovalerate aldolase, and pyruvate dehydrogenase complex. Similarly, acetyl-CoA will then enter the TCA cycle and participate in biomass synthesis or accumulation of other metabolites [17,25]. Another proposed pathway of biodegradation of PS starts from the side-chain cleavage of PS. Monooxygenases or aromatic ring hydroxylases are potential candidates to break the aromatic ring of PS. However, the detailed degradation pathway and involved enzymes have not still been revealed [17].

The most used bacteria for PS degradation are genera *Bacillus* [5,14,26,27], *Pseudomonas* [26], *Paenibacillus* [28], and *Rhodococcus* [29,30], while *Aspergillus* [5] and *Curvularia* [31] are the most investigated genera of fungi. Asmita et al. [5] reported PS weight loss of 20.0 and 5.0% by *Bacillus subtilis* and *Pseudomonas aeruginosa*, respectively, which were cultured in nutrient broth. In the other case, bacteria were cultured in Bushnell–Hass broth, and the weight loss of PS was higher (58.8%) for *Bacillus cereus*, but for *Pseudomonas aeruginosa,* weight loss was not observed. This indicates that providing a suitable nutrient medium for bacteria is important to enhance the biodegradation process. Auta et al. [14] investigated the biodegradation of PS microplastics by *Bacillus cereus* for 40 days. They reported a weight loss of PS of 7.4%. Moreover, a slightly higher weight loss was observed by Mohan et al. [26]. They investigated HIPS film degradation using *Bacillus* spp. and *Pseudomonas* sp. The weight loss percentage of HIPS film obtained after treatment with *Bacillus* spp. was significant at 23.7% and was less than 10.0% with *Pseudomonas* sp. Moreover, Kumar et al. [32] reported a PS weight loss of 34.0% by *Bacillus paralicheniformis* G1 after 60 days of exposure. However, the exposure of PS to *Rhodococcus ruber* for up to 8 weeks resulted in a small reduction in PS weight loss (0.8%). Mor and Sivan [30] demonstrated the high affinity of *Rhodococcus ruber* to PS, which led to biofilm formation and, presumably, induced partial biodegradation. Furthermore, *Pseudomonas* sp. was tested for PS degradation, and according to Kim et al. [33], colonies of *Pseudomonas* sp. were observed on the PS film after 30 days. Scanning electron microscopy analysis (SEM) revealed smoother edges and holes in the PS film. The viability and proliferation of *Pseudomonas* sp. DSM 50071 on the surface PS suggests that PS can be used as an energy resource and cellular component when no other alternative carbon sources are available. In addition, the study by Motta et al. [31] tested pretreated PS films. In order to induce changes in the structure of PS and thus facilitate the mechanism by which microorganisms can take up the carbon contained in the polymer, PS was first subjected to novel chemical oxidation treatments that can convert the polymer chains into more oxidized compounds with presumably lower molecular weights. These treatments trigger a series of physical and chemical changes that lead to the formation of carbonyl and hydroxyl groups. In one treatment, the oxidant was used alone (sample 1); in another, a transition metal complex was added to the oxidant (sample 2); and in the third, an inorganic acid was added to the oxidant (sample 3). In all treatments, the polymers were exposed to the oxidant for 2 h at room temperature and sterilized with UV radiation before incubation with the fungi. After pretreatment, the films were placed on plates containing Sabouraud agar on which the fungus *Curvularia* sp. was growing. After 4 weeks of exposure, *Curvularia* sp. began to colonize the surface of the film pretreated with the oxidizing agent. However, after a longer period of time (9 weeks), *Curvularia* sp. completely colonized the surface of the pretreated PS. Colonization of the non-pretreated PS did not occur. These results suggest that improvement in biodegradation is possible by pretreating the MP as well as by longer exposure time. Furthermore, acceleration of biodegradation can be achieved by co-metabolism. In this process, primary substrates (e.g., glucose) are added to the polluted medium to stimulate the microorganisms to produce degrading enzymes. However, this does not always have a positive effect on decomposition. For example, Shabbir et al. [34] reported that the addition of glucose increased the biodegradation of MP by the periphyton biofilm for all MP (from 9.52–18.02%, 5.95–14.02%, and 13.24–19.72% for PP, PE, and PET) after 60 days, respectively, while a mixture of peptone and glucose and peptone together had an inhibitory effect. Accordingly, there are a variety of factors that influence the biodegradation of MP. The study of key biodegradation factors can be achieved by the Taguchi experimental design, which allows a minimal number of experiments, saving time and resources. These are arrays selected according to the number of factors and levels. The influencing factors are arranged in orthogonal arrays, and the levels on which they are examined vary. The Taguchi method is most suitable for studying processes where there are few interactions between factors; some factors are statistically significant, and there is an average number of factors, such as 3 to 50 [35]. However, the study of optimal conditions is usually conducted by changing one variable while all other variables remain fixed in a given set of conditions. To find the correct optimum, possible interactions between variables should be considered. On the other hand, factorial experimental design can be used to estimate the interactions of possible influencing parameters on efficiency with a limited number of planned trials [36]. In addition, factorial experimental designs allow the investigation of the joint effect of factors on a response. Full factorial experiments consist of all possible combinations of values for all factors [37]. This experimental design is also commonly used in bioremediation studies [36,38].

This study investigated the significant factors affecting the biodegradation of MP-PS particles by bacteria *Bacillus cereus* and *Pseudomonas alcaligenes*. Experiments were conducted using the Taguchi method, and seven factors were investigated at two levels. After 30 days of experiments, the statistically significant factors for biodegradation were determined using colony-forming units (CFU) as the response parameter. Subsequently, biodegradation experiments were conducted with the obtained significant factors using the full factorial design to determine the optimal conditions for the biodegradation of PS by the two mentioned bacteria.

## 2. Materials and Methods

### 2.1. Microplastics Preparation

MP were obtained by grinding plastic disposable accessories such as spoons for PS. First, these materials were cut into smaller pieces with scissors and then ground in a cryo-mill (Retsch, Haan, Germany) with liquid nitrogen and dried in the air for 48 h at room temperature. Next, obtained particles were sieved on stainless steel screens (RX-86-1 Sieve shaker, W.S. Tyler, Mentor, OH, USA) to obtain particles in size ranges: 500–700 µm, 300–500 µm, and 100–300 µm. After sieving, the MP particles were stored in glass bottles. Before the experiments, MP particles were sterilized in 100 mL flasks containing 70% ethanol for 10 min on a rotary shaker (Heidolph unimax 1010, Heidolph incubator 1000, Schwabach, Germany) at 160 rpm. The particles were separated from the ethanol by vacuum membrane filtration (through cellulose nitrate 0.45 µm sterile filter (ReliaDisc^TM^, Ahlstrom-Munksjö, Helsinki, Finland)) and additionally washed with sterile deionized water.

### 2.2. Bacterial Cultivation

**Figure 1:**
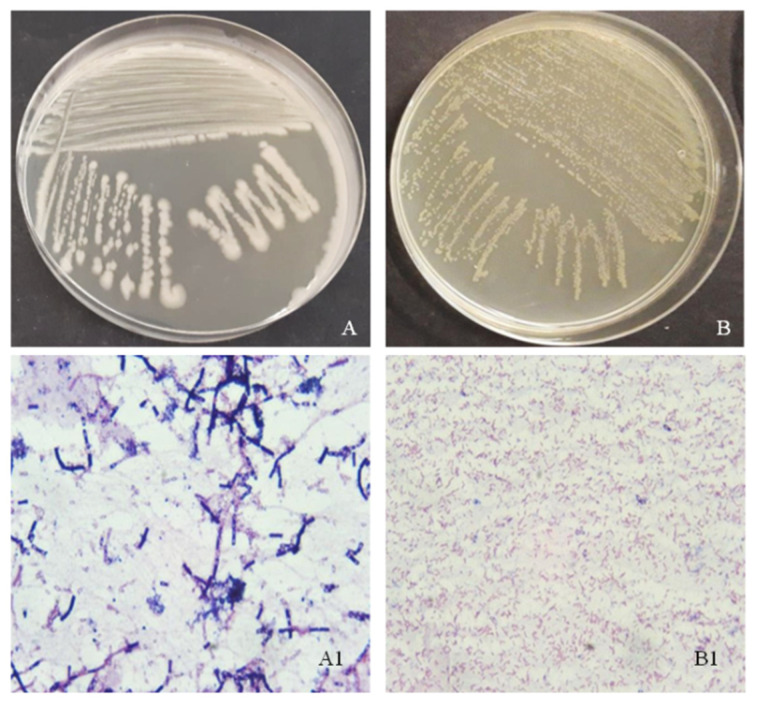
Bacteria (**A**) *Bacillus cereus* and (**B**) *Pseudomonas alcaligenes* cultivate on nutrient agar by streak plate method. Microphotos of bacterial Gram-stained smears, (**A1**) *Bacillus cereus,* and (**B1**) *Pseudomonas alcaligenes* at the microscope magnification of 1000×.

The bacteria *Bacillus cereus* and *Pseudomonas alcaligenes* were isolated from environmental samples in which MP particles were present, from activated sludge (municipal wastewater treatment plant, Vrgorac, Croatia) and river sediment (Kupa, Croatia). In total, 100 mL of activated sludge and river sediment were placed in 250 mL sterilized Erlenmeyer flasks and shaken for 24 h at room temperature on the thermostatic rotary shaker at 160 rpm. After 24 h, the colony-forming units (CFU) of bacteria on the general-purpose medium (nutrient agar (NA)) were determined using the pour plate method according to Briški et al. [39]. For plate counting, a dilution series (0.9% mass aqueous NaCl solution) was prepared from each sample. The plates were incubated at 80% relative humidity and 37 °C to culture the bacteria. After incubation, the number of colonies on the agar plates was determined. Bacterial colonies that were morphologically distinct and dominated on the nutrient agar plates were collected and transferred to the nutrient agar plates and incubated at 37 °C for 24–48 h. After pure isolates were obtained, they were stored in slants for characterization. The isolated bacteria were Gram-stained (Figure 1) [40]. After Gram staining, a series of biochemical tests known as API (Analytical profile index, bioMérieux^®^, Lyon, France) were performed. Gram-negative bacteria were identified using API strip 20 E (Analytical Profile Index, bioMérieux^®^, Lyon, France). The final step of bacterial identification was matrix-assisted laser desorption/ionization time-of-flight mass spectrometry (Microflex LT MALDI-TOF MS, Bruker Daltonics, Bremen, Germany), which is based on protein identification of pulsed single ionic analytes (pure microbial culture) coupled with a TOF measurement mass analyzer, and the exact protein mass was determined. Cultures were set on the pre-cultivation in the mineral media [41] for 24 h before experiments in order to achieve the growth’s log phase. The optical density (*OD*) of bacterial suspension was measured on a spectrophotometer (DR/2400 Portable Spectrophotometer, Hach, Loveland, CO, USA) at *λ* = 600 nm, and CFU was determined by the decimal plate method [42].

### 2.3. Biodegradation Experiments

Biodegradation of MP-PS particles was investigated by the Gram-positive bacterium *Bacillus cereus* and the Gram-negative bacterium *Pseudomonas alcaligenes*, respectively. The mentioned microorganisms were isolated from environmental samples where MP particles were found and adapted to the above conditions. Therefore, it can be assumed that the bioaugmentation of the autochthonous microorganisms into the system will accelerate the biodegradation process. The research was divided into two parts: preliminary experiments according to the Taguchi design (P1 and P2) and the main experiments by full factorial design (M1 and M2). All experiments were carried out in duplicate.

The experiments (preliminary and main) were conducted in 250 mL Erlenmeyer flasks with a working volume of 100 mL for 30 days at the thermostatic rotary shakers (Heidolph unimax 1010, Heidolph incubator 1000, Schwabach, Germany). The temperatures and agitation speeds on the rotary shakers were set according to the experimental plan. The flasks contained mineral medium (composition according to [41]), bacterial suspension (*Bacillus cereus* or *Pseudomonas alcaligenes*), and MP-PS. The control flasks were set for the purpose of monitoring bacterial growth and contain mineral medium and bacterial suspension without MP-PS particles.

#### 2.3.1. Preliminary Experiments

Preliminary experiments were performed according to the L_8_ orthogonal array listed in Table 1 and obtained by the Taguchi design (7 factors at 2 levels). Significant factors (pH-value, temperature, *T*, MP size, concentration of MP, *γ*_MP_, agitation speed, optical density of bacterial suspension, *OD*, and addition of glucose, *γ*_GLU_) that can influence the biodegradation process of MP-PS by *Bacillus cereus* and *Pseudomonas alcaligenes* were investigated. Experiments (presented in Table 1) were marked as P1 (1-1 to 1-8) and P2 (2-1 to 2-8) for MPs-PS biodegradation by *Bacillus cereus* and *Pseudomonas alcaligenes*, respectively. In experiments with *Bacillus cereus*, CFU were 5.5 × 10^6^ cells/mL and 2.6 × 10^7^ cells/mL at optical densities of 0.1 and 0.5, respectively. In experiments with *Pseudomonas alcaligenes*, CFU values were 4.5 × 10^7^ cells/mL and 3.5 × 10^8^ cells/mL at optical densities of 0.1 and 0.5, respectively.

Analyses of key process parameters, by contribution, for PS biodegradation by *Bacillus cereus* and *Pseudomonas alcaligenes* were determinate using the logarithmic CFU value (log CFU) as a response parameter. The influence of each of the above factors was assessed using the L_8_ orthogonal array method, and the factors with the greatest contribution by analysis of variance were determined (ANOVA). Statistical significance of the factors’ effects was considered at a 95.0% confidence level. Calculations and statistical analyses were performed using Design-Expert 10.0, Stat-Ease, Minneapolis, MN, USA.

#### 2.3.2. Main Experiments

Preliminary experiments pointed out three factors with the highest contribution to biodegradation. Based on the results of the preliminary experiments, the authors designed two new sets of experiments (M1 and M2 for *Bacillus cereus* and *Pseudomonas alcaligenes*, respectively) to determine the optimal conditions for MP-PS biodegradation. The experiments were designated following the full factorial methodology with 3 factors at 3 levels, resulting in a total of 27 experiments each (Table 2). M1 experiments included MP concentration (*γ*_MP_), MP size, and agitation speed as factors. The pH-value of the mineral medium (7.323), temperature (25 ± 0.2 °C), and optical density (*OD* = 0.3) were constant. In the case of M2, the factors were optical density, agitation speed, and size of MP particles; the pH-value of the mineral medium (7.323), temperature (25 ± 0.2 °C), and concentration of MP (500 mg/L) were constant in this set of experiments. In all experiments (M1 and M2), glucose was not added to the media. Calculations and statistical analyses were performed using Design-Expert 10.0, Stat-Ease, Minneapolis, MN, USA.

During these experiments, *OD* and CFU were measured in order to analyze bacterial growth. After 30 days of the biodegradation process, the optimal conditions for MP-PS biodegradation by *Bacillus cereus* and *Pseudomonas alcaligenes* were determinate using the log CFU as a response parameter.

Additional analyses of the aqueous phase during the experiments were also performed to verify the biodegradation process and to get a better insight into the whole process. Total carbon (TC), total organic carbon (TOC), and inorganic carbon (IC) were determined (TOC-VCSH, Shimadzu, Japan). Released additives in the aqueous phase were estimated by LC/MS analysis (LC-MS 2020, Shimadzu, Japan).

At the end of the experiments, the MP-PS particles were separated from the aqueous phase by vacuum membrane filtration (and washed with sterile deionized water). The ecotoxicity of the aqueous phase (Lumistox 300, Dr. Lange GmbH, Düsseldorf, Germany) with the marine bacterium *Vibrio fischeri* [43] was conducted.

MP-PS particles were analyzed before and after biodegradation by FTIR-ATR spectroscopy (Spectrum One, PerkinElmer, Waltham, MA, USA).

### 2.4. Response Surface Modeling

Response surface modeling (RSM) was applied to define the influence of the concentration of MP particles (*X*_1_), particles size (*X*_2_), and agitation speed (*X*_3_) on the logarithmic number of the living cells (log CFU) of *Bacillus cereus* in M1 experiments. For M2 experiments, it was necessary to define the influence of agitation speed (*X*_1_), particles size (*X*_2_), and *OD* (*X*_3_) on the logarithmic number of the living cells (log CFU) of *Pseudomonas alcaligenes*. For that purpose, the MP size intervals were replaced by corresponding average values: 200, 400, and 600 µm. Two polynomials of various complexities were applied to describe the response surface. The models were presented by Equations (1) and (2).

log CFU = *a*_0_ + *a*_1_*X*_1_ + *a*_2_*X*_2_ + *a*_3_*X*_3_ (1)

log CFU = *a*_0_ + *a*_1_*X*_1_ + *a*_2_*X*_2_ + *a*_3_*X*_3_ + *a*_4_*X*_1_·*X*_2_ + *a*_5_*X*_1_·*X*_3_ + *a*_6_*X*_2_·*X*_3_ + *a*_7_*X*_1_^2^ + *a*_8_*X*_2_^2^ + *a*_9_*X*_3_^2^ (2)

Letter *a*, used in these models, represents model coefficients. MODEL I (Equation (1)) contains linear contributions of the concentration/agitation speed, particle size, and agitation speed/optical density for M1 and M2 experiments, respectively. MODEL II (Equation (2)) is, in fact, MODEL I upgraded by the interaction terms (*X*_1_·*X*_2_, *X*_1_·*X*_3_, and *X*_2_·*X*_3_) as well as by quadratic terms (*X*_1_^2^, *X*_2_^2^, and *X*_3_^2^). Calculations and analyses were performed using Design-Expert 10.0, Stat-Ease, Minneapolis, MN, USA.

## 3. Results and Discussion

### 3.1. Preliminary Experiment

In the preliminary experiments, seven factors (*T*, pH-value, *γ*_MP_, *γ*_GLU_, agitation speed, size of MP, and *OD*) were examined on two levels according to the Taguchi design, Table 1. According to the response parameter, log CFU (Table 3), the statistically significant factors for the biodegradation of MP-PS by *Bacillus cereus* were the size of MP (48.52%), *γ*_MP_ (5.03%), and agitation speed (41.31%). From the results according to the contribution, the agitation speed and the size of MP-PS had the greatest influence on the biodegradation of MP-PS by *Bacillus cereus*. This is not surprising, as the smaller particle size and larger surface area contribute to the bacterial colonization of the MP-PS particles, and colonization is considered the first necessary step for biodegradation [25]. In addition, the rotary shaker’s agitation speed maintains the dissolved oxygen concentration, which is essential for biochemical reactions [44]. Furthermore, increased oxygen concentration accelerates the degradation of the polymer [45]. According to the oxygen demand, *Bacillus cereus* is a facultative anaerobe that can adapt to and grow in anoxic conditions [46]. Moreover, the presence of higher concentrations of MP-PS can negatively affect the biodegradation process due to its toxic effects, such as reducing the efficiency of the photosynthesis process and damaging the cells of microorganisms [47]. Over time, they can release various additives (plasticizers, stabilizers, pigments, fillers, and flame retardants) that have been shown to have a toxic effect on organisms [48,49]. In addition, this rod-shaped bacterium forms endospores that make it more resistant to extreme environmental conditions and enable its growth, adaptability, and survival [46].

According to Table 3, significant factors for the biodegradation of MP-PS by *Pseudomonas alcaligenes* were agitation speed (53.42%), size of MP-PS (14.48%), and *OD* (13.91%). However, the agitation speed and size of MP-PS had a higher impact on the biodegradation process due to higher percentages of contribution. As mentioned above, the agitation speed is important to ensure the dissolved oxygen concentration in the system. Apart from this effect, it also allows homogenization and bioavailability of particles to bacteria. Indeed, *Pseudomonas alcaligenes* is an aerobic bacterium, which means that the dissolved oxygen concentration is essential for its growth [50]. This confirms our results, as the better biodegradation of PS by *Pseudomonas alcaligenes* occurred at the higher tested agitation speed (200 rpm). *Pseudomonas alcaligenes* is a Gram-negative, rod-shaped bacterium commonly used in bioremediation for the degradation of polycyclic aromatic hydrocarbons [51]. One of the many factors that influence the biodegradation of MP-PS is particle size; a larger particle size means a smaller surface area, which directly reduces the possibility of colonization (biofilm formation) of MP-PS by bacteria [25]. In addition, *OD* represents the number of live and dead bacterial cells and is a useful and technically simple parameter to indicate bacterial growth. A higher *OD* value means a higher number of bacterial cells, which consequently increases the efficiency of the biodegradation process. However, CFU determination is a better indicator of bacterial growth.

### 3.2. Main Experiment

Optimal conditions for biodegradation of MP-PS were determined from the main experiments, M1 and M2, for *Bacillus cereus* and *Pseudomonas alcaligenes*, respectively.

**Figure 2:**
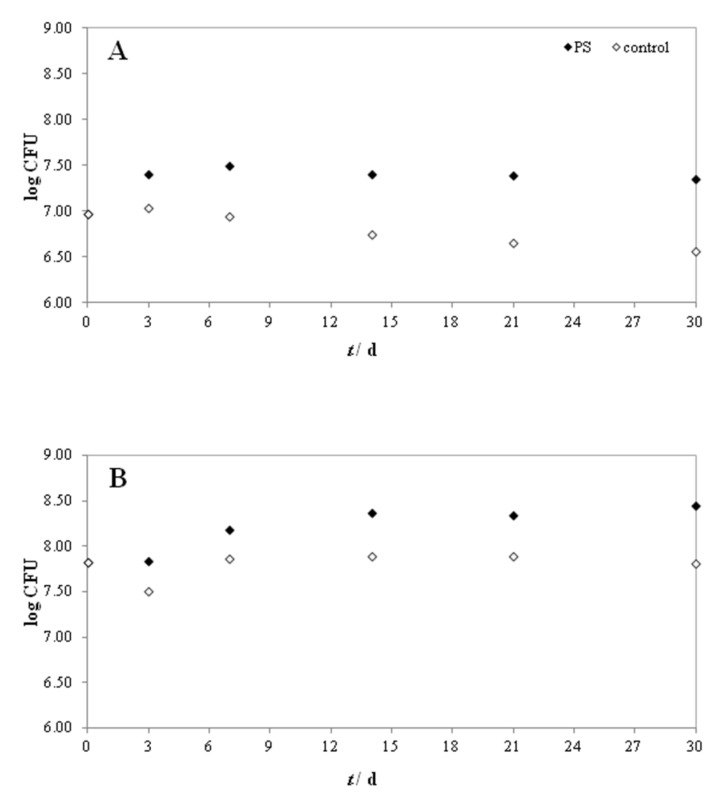
Changes of log CFU of (**A**) *Bacillus cereus* in experiment M1-24 and (**B**) *Pseudomonas alcaligenes* in experiment M2-23 during 30 days of MP-PS biodegradation.

To monitor bacterial growth, CFU was determined during the biodegradation of MP-PS (Figure 2). CFU as the number of live bacterial cells was increased until days 7 and 14 of the experiment M1-24 and M2-23, respectively. CFU of *Bacillus cereus* (experiment M1-24) increased from the initial value (9.3 × 10^6^ cells/mL) at the beginning of the experiment to 3.2 × 10^7^ cells/mL on the 7th day. The same trend was observed in M2-23 for *Pseudomonas alcaligenes*, which showed a higher increase (from an initial CFU of 6.5 × 10^7^ cells/mL to 2.3 × 10^8^ cells/mL on day 14). After the exponential phase of bacterial growth, the stationary phase occurred (Figure 2A,B) in which the number of live and dead bacterial cells is equal. On the last day (day 30) of the experiments, the CFU was 2.2 × 10^7^ cells/mL and 2.8 × 10^8^ cells/mL for *Bacillus cereus* and *Pseudomonas alcaligenes*, respectively. Accordingly, the studied conditions were favorable for the growth and multiplication of the bacteria due to the possible production of degradation products. In addition, the CFU values of the control were lower compared to the samples containing MP-PS. This suggests that bacteria have adapted to the conditions with MP-PS and are likely using MP-PS as a carbon source for growth. This ability of bacteria of the genera *Bacillus* and *Pseudomonas* has been investigated by other researchers [14,26,33].

**Figure 3:**
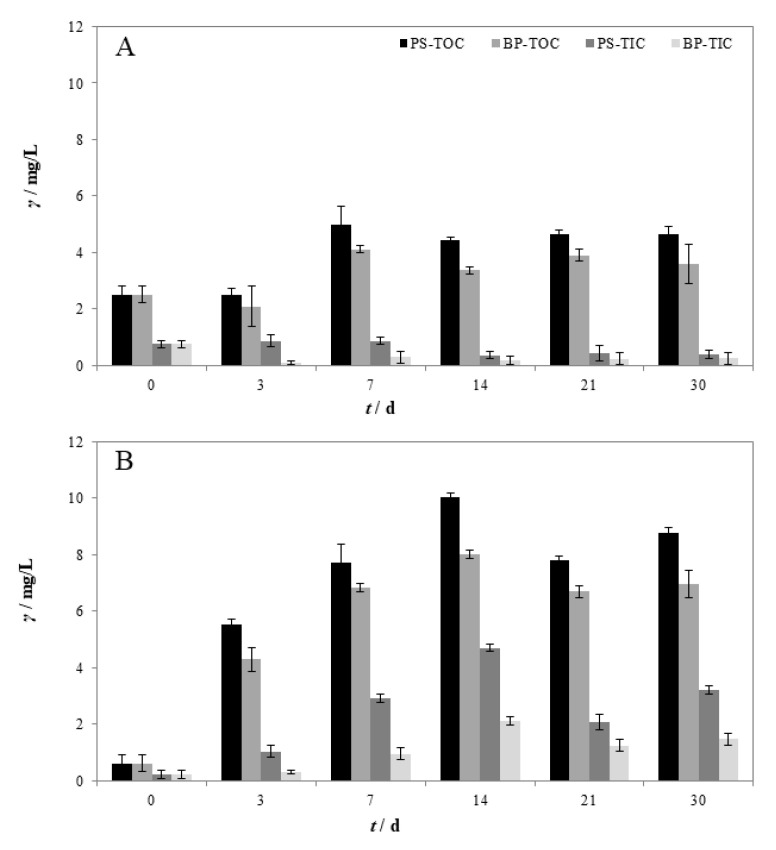
Changes in concentration (*γ*) of total organic carbon (TOC) and total inorganic carbon (TIC) for blank (BP) and sample (MP-PS) during 30 days of exposure to: (**A**) *Bacillus cereus* in experiment M1-24 and (**B**) *Pseudomonas alcaligenes* in experiment M2-23.

**Figure 4:**
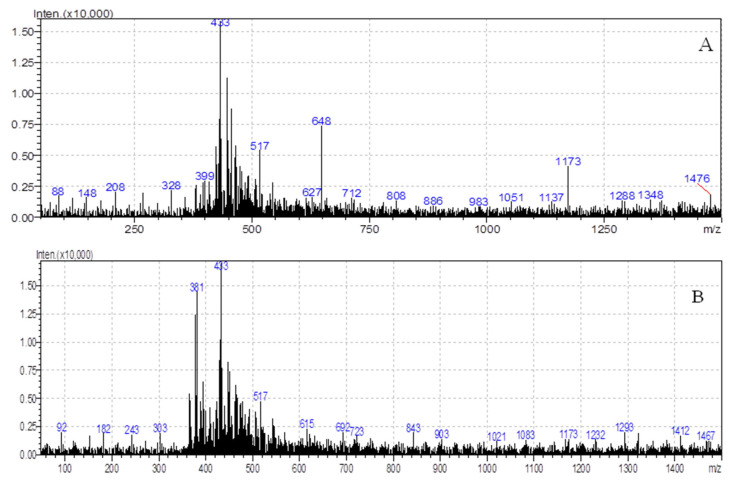
LC/MS analysis of aqueous phase for biodegradation process of MP-PS by (**A**) *Bacillus cereus* in experiment M1-24 and (**B**) *Pseudomonas alcaligenes* in experiment M2-23.

The same trend as CFU was observed in the changes of TOC and TIC values of the aqueous phase in M1-24 (Figure 3A) and M2-23 (Figure 3B). The TOC and TIC values increased until the 7th day. After the 7th day and until the end of the experiment, the TOC and TIC values had not changed significantly. This is consistent with the growth stages of *Bacillus cereus* shown in Figure 2A. In experiment M2-23, TOC and TIC values increased until day 14. These changes were also consistent with the changes in CFU of *Pseudomonas alcaligenes* during the 30-day biodegradation of PS. However, TOC and TIC concentrations in the blank (BP) also increased, indicating the lysis of bacterial cells [27,52]. Compared to BP, TOC and TIC values were higher in samples with MP-PS particles, indicating the utilization of MP-PS by bacteria. Higher TOC and TIC values were observed for samples with *Pseudomonas alcaligenes*, correlating with higher CFU values for this bacterium. This indicates more efficient biodegradation of MP-PS by *Pseudomonas alcaligenes* than *Bacillus cereus*. An increase in TOC concentration in the sample may indicate the production of degradation products and/or the release of additives from the surface MP-PS. The increase in TOC concentration in the sample correlates with an increase in TIC levels, indicating that biodegradation has occurred [52]. In general, the TOC content of polluted water decreases during biodegradation [53]. This trend usually correlates with the increase in TIC since CO_2_ is a product of mineralization [54]. However, during the biodegradation of water containing solid plastic particles, the TOC content may not decrease due to the plastic particles’ release of additives or/and synthetic polymer analogs. These compounds may be mineralized to CO_2_, which is reflected in an increase in the TIC value. This is consistent with the LC/MS analysis results of the aqueous phases (Figure 4).

During the exposure of *Bacillus cereus* and *Pseudomonas alcaligenes* to MP-PS particles, additives that were added to the material during production may be released in the aqueous phase, and that was monitored by a flow injection LC/MS analysis. At the beginning of the experiment, no peaks were recorded on the chromatogram. However, on the 7th day, peaks appeared in the chromatogram (Figure 4), indicating the release of the additive from the surface MP. LC/MS analyses of the aqueous phase for the M1-24 experiment (Figure 4A) indicate the presence of [M+H]+ product at *m*/*z* peak of 328 that may represent triphenyl phosphate, flame retardant in plastics [55]. Triphenyl phosphate is a commonly used commercial chemical additive that is classified as an organophosphate flame retardant and poses a potential toxic risk to aquatic organisms [56]. In the sample of M2-23, an antioxidant additive butylated hydroxytoluene was assumed at an *m*/*z* ratio of 243 (Figure 4B) in the form [M+Na]+ [55]. Butylated hydroxytoluene, as a phenolic compound, is one of the frequent antioxidants used to protect plastics against oxidation (during their exposure to heat and light) [57]. Generally, according to Ho et al. [4] and De-la-Torre et al. [58], various additives such as antioxidants, UV stabilizers, processing lubes, antistats, and flame retardants may be incorporated into MP-PS. These compounds improve MP-PS properties but cause serious ecotoxicological concerns for the water environment.

The ecotoxicity test was performed with *Vibrio fischeri* for the aqueous phases of experiments M1-24 and M2-23, which are shown in Table 4. The inhibition value (*INH*) of the aqueous phase for M2-23 was higher than the *INH* value for M1-24, indicating higher toxicity of the sample obtained after the biodegradation of PS by *Pseudomonas alcaligenes*. The lower EC_20_ value in M1-24 also indicates higher toxicity of the mentioned sample. These results confirm the previously mentioned findings regarding the higher CFU, TOC, and TIC values in M2-23 than in M1-24 and the released additives. The aqueous phase of M2-23 contained some degradation products and/or additives that may be toxic to aquatic organisms. However, these obtained toxicity values are relatively low due to the not possible estimation of *EC*_50_.

**Figure 5:**
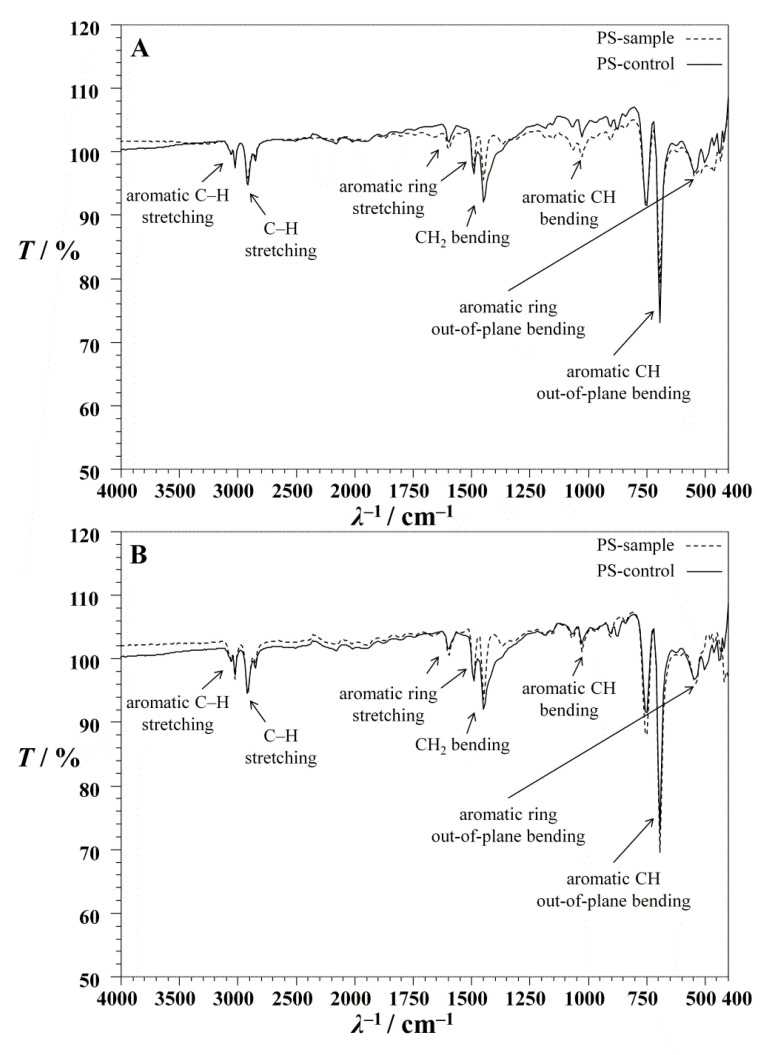
FTIR-ATR spectroscopy of MP-PS particles before (MP-PS control) and after (MP-PS sample) biodegradation by: (**A**) *Bacillus cereus* in experiment M1-24 and (**B**) *Pseudomonas alcaligenes* in experiment M2-23.

For the purpose of confirming MP-PS biodegradation, FTIR-ATR analyses were carried out. Characteristic MP-PS peaks at 3024, 2847, 1601, 1492, 1451, 1027, and 694 cm^−^^1^ [59] are in Figure 5. The peak of 3024 cm^−^^1^ is specific for aromatic C−H stretching while stretching of the other C−H groups is detected at 2847 cm^−^^1^. Wavenumbers 1601 and 1492 cm^−^^1^ are related to aromatic ring stretching. The bending of the CH_2_ group occurs at 1451 cm^−^^1^, while the bending of the aromatic C−H groups has characteristic peaks at 1027 and 694 cm^−^^1^ [59]. All characteristic peaks decreased their intensities after biodegradation by *Bacillus cereus* (M1-24) (Figure 5A). The decrement in intensities of characteristic FTIR-ATR peaks was noticed after treatment by *Pseudomonas alcaligenes* (M2-23), as well (Figure 5B). The peak between 1000 to 750 cm^−^^1^, representing C−H groups, disappeared in both spectra, while the new peak at approximately 1395 cm^−^^1^ appeared; Subramani and Sepperumal [60] noted the appearance of the same peak during the biodegradation of PS foam by *Pseudomonas* sp. During the oxidation process, functional groups such as hydroxyl or carbonyl groups could be formed via β-oxidation, which are known to be used in the TCA cycle or in the energy metabolism of bacteria, thereby increasing hydrophilicity [61,62]. A characteristic weak peak at 1715 cm^−1^ was detected in both spectra, indicating the formation of carbonyl groups (−C=O). Therefore, the increased number of oxygen atoms on the plastic surface in areas exhibiting bacterial growth is direct evidence of PS degradation [16].

**Figure 6:**
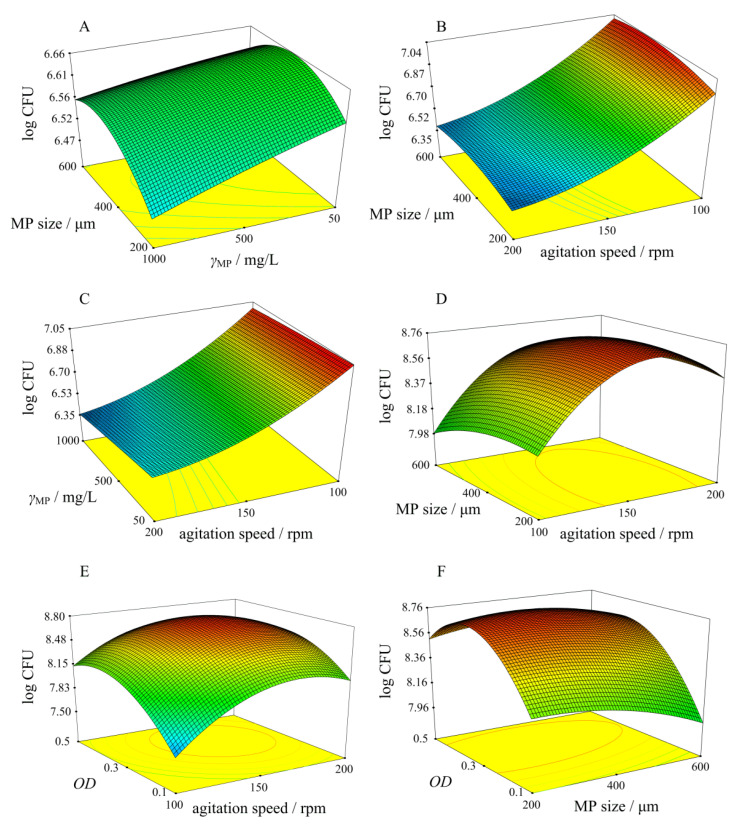
Response surfaces obtained for MP-PS biodegradation by *Bacillus cereus* (**A**–**C**) and by *Pseudomonas alcaligenes* (**D**–**F**) in main experiments by full factorial design.

According to significant parameters (Table 3), the optimal conditions for MP-PS biodegradation were investigated by full factorial design. Three factors were examined at three levels (Table 5), and response surfaces (Figure 6) were created to determine the effect of the factors on changes in log CFU. As can be seen in Figure 6A, the concentration and size of MP-PS have no significant effect on the growth of bacteria (CFU). In contrast, the living colonies of *Bacillus cereus* (CFU) increased with decreasing agitation speed and MP-PS concentration (red parts of response surfaces in Figure 6B,C). These results indicate a greater influence of agitation speed on the biodegradation of MP-PS by *Bacillus cereus*. In the case of biodegradation of MP-PS by *Pseudomonas alcaligenes*, the number of bacteria colonies (CFU) increased with increasing agitation speed and at a particle size of PS = 400 µm (Figure 6D). Figure 6E shows an increase in the number of live bacteria colonies with increasing agitation speed and *OD* = 0.3. According to the red area in Figure 6F, the highest log CFU was observed at *OD* = 0.3, and the mean investigated particle size of MP-PS (200 µm). This indicates a high effect of all three investigated factors on CFU values, which was confirmed by ANOVA analysis (Table 5).

The analysis of the developed mathematical models revealed information about the biodegradation of MP-PS by *Bacillus cereus*, Table 5. Two models (linear and quadratic) were used to describe the experimental data. MODEL I showed the agitation speed of the rotary shaker (term *X*_3_) as the influential factor. The associated coefficient of determination had a relatively low value (*R*^2^ = 0.8616) compared to more complex polynomials such as MODEL II. Following the highest value of *R*^2^ for MODEL II, this model was the most statistically significant in describing the biodegradation of MP-PS particles. Statistical analysis of MODEL II pointed out that the agitation speed of the rotary shaker (terms *X*_3_ and *X*_3_^2^) was the only factor influencing the MP-PS biodegradation. Moreover, statistical analysis of the biodegradation experiments with *Pseudomonas alcaligenes* shows that MODEL II proved to be the best model to describe this system (Table 5). The higher values of *R*^2^ and *R*^2^_adj_ for MODEL II suggest that the statistical analysis is much closer to the experimental data, indicating the greater significance of MODEL II compared with MODEL I. In this case, the large, influential factors were agitation speed, size of MP-PS particles, *OD*, the interaction term of agitation speed and *OD*, as well as quadratic terms of agitation speed and *OD* (terms *X*_1_, *X*_2_, *X*_3_, *X*_1_· *X*_3_, *X*_1_^2^, and *X*_3_^2^, respectively).

Overall, optimal biodegradation conditions are represented in Table 6. These results are in agreement with previously explained response surfaces and statistical analysis. Accordingly, the most efficient biodegradation of MP-PS by *Bacillus cereus* can be obtained at low MP-PS concentration and some average MP-PS particle size at 100 rpm. On the other hand, an average MP-PS particle size at a higher agitation speed and the average value of *OD* was the most suitable conditions for *Pseudomonas alcaligenes*. From the determined optimal conditions for biodegradation, the optimal particle size of MP is medium in both cases, which is surprising considering that the smaller particle size has a larger surface area. Thus, the bioavailability of the particles for biodegradation is better. However, according to previous studies, the toxic effect of MP increases with decreasing particle size [63,64].

## 4. Conclusions

This research investigated the biodegradation of MP-PS particles by the bacteria *Bacillus cereus* and *Pseudomonas alcaligenes*. There are many factors that influence the biodegradation process of MP-PS. Hence, it is necessary to ensure optimal conditions for bacteria to stimulate bacteria for MP-PS biodegradation. Accordingly, the key factors that significantly influence these two bacteria’s biodegradation of MP-PS were investigated. After this step, the determination of optimal conditions for MP-PS biodegradation was studied. In conclusion, the agitation speed of the rotary shaker plays a key role during MP-PS biodegradation for both bacteria. Furthermore, additional analyses provided a better understanding of the biodegradation process. Bacterial growth was monitored by determining the CFU; CFU changes were correlated with TOC values; an increase in TOC was observed due to the deterioration of PS structure and release of additives, which were correlated with LC/MS results. According to the *m*/*z* peaks, the flame-retardant triphenyl phosphate and the antioxidant butylated hydroxyl toluene were noticed. The increase in TIC values indicates the formation of CO_2_, which is a product of biodegradation. By FTIR-ATR spectroscopy, the deterioration of the MP-PS structure was confirmed due to the carbonyl group formation in both used bacteria. Overall, the obtained results indicate a higher biodegradation potential of MP-PS for *Pseudomonas alcaligenes*. However, *Bacillus cereus* and *Pseudomonas alcaligenes* are suitable choices for biodegrading MP-PS particles due to their great adaptability to various conditions. Future research should be focus on investigating these optimal biodegradation conditions.

## Figures and Tables

**Table 1:** Taguchi L_8_ orthogonal array for each factor at 2 levels.

Experiment No.	pH/-	*T*/°C	MP Size/µm	*γ*_MP_/mg/L	Agitation Speed/rpm	*OD*/-	*γ*_GLU_/mg/L
P1-1; P2-1	8	15	600	1000	100	0.5	0
P1-2; P2-2	6	15	200	50	100	0.1	0
P1-3; P2-3	6	15	200	1000	200	0.5	100
P1-4; P2-4	8	15	600	50	200	0.1	100
P1-5; P2-5	6	25	600	50	100	0.5	100
P1-6; P2-6	8	25	200	1000	100	0.1	100
P1-7; P2-7	6	25	600	1000	200	0.1	0
P1-8; P2-8	8	25	200	50	200	0.5	0

**Table 2:** Full factorial experimental design for MP-PS biodegradation process by bacteria *Bacillus cereus* and *Pseudomonas alcaligenes* at 3 levels.

*Bacillus cereus*				*Pseudomonas alcaligenes*			
Experiment No.	*γ*_MP_/mg/L	MP Size/µm	Agitation Speed/rpm	Experiment No.	Agitation Speed/rpm	MP Size/µm	*OD*/-
M1-1	1000	400	100	M2-1	200	400	0.1
M1-2	50	400	200	M2-2	100	400	0.5
M1-3	1000	200	150	M2-3	200	200	0.3
M1-4	50	200	100	M2-4	100	200	0.1
M1-5	500	400	100	M2-5	150	400	0.1
M1-6	50	600	150	M2-6	100	600	0.3
M1-7	50	600	100	M2-7	100	600	0.1
M1-8	500	200	100	M2-8	150	200	0.1
M1-9	500	600	100	M2-9	150	600	0.1
M1-10	1000	200	200	M2-10	200	200	0.5
M1-11	1000	200	100	M2-11	200	200	0.1
M1-12	50	200	150	M2-12	100	200	0.3
M1-13	500	400	200	M2-13	150	400	0.5
M1-14	500	400	150	M2-14	150	400	0.3
M1-15	1000	600	150	M2-15	200	600	0.3
M1-16	50	600	200	M2-16	100	600	0.5
M1-17	50	200	200	M2-17	100	200	0.5
M1-18	1000	600	200	M2-18	200	600	0.5
M1-19	1000	600	100	M2-19	200	600	0.1
M1-20	500	600	150	M2-20	150	600	0.3
M1-21	500	600	200	M2-21	150	600	0.5
M1-22	1000	400	200	M2-22	200	400	0.5
M1-23	1000	400	150	M2-23	200	400	0.3
M1-24	50	400	100	M2-24	100	400	0.1
M1-25	50	400	150	M2-25	100	400	0.3
M1-26	500	200	150	M2-26	150	200	0.3
M1-27	500	200	200	M2-27	150	200	0.5

**Table 3:** Results of preliminary experiments designed by the Taguchi method for biodegradation of MP-PS by *Bacillus cereus* and *Pseudomonas alcaligenes*.

	*Bacillus cereus*			*Pseudomonas alcaligenes*		
Factors	*γ*_MP_	MP Size	Agitation Speed	Agitation Speed	MP Size	*OD*
Sum of Squares	0.07	0.68	0.58	1.88	0.51	0.49
*DF* *	1	1	1	1	1	1
*F*-value	128.82	1243.26	1058.59	46.93	12.72	12.22
*p*-value	0.002	0.000	0.000	0.006	0.038	0.040
Contribution/%	5.03	48.52	41.31	14.48	14.48	13.91

* *DF* = degree of freedom.

**Table 4:** Inhibition of aqueous phase (*INH*) and *EC* values (*EC*_20_) obtained by ecotoxicity tests for the aqueous phase in experiments M1-24 and M2-23 by *Vibrio fischeri*.

Experiment No.	*INH*/%	*EC*_20_/%
M1-24	40.11	28.57
M2-23	46.79	27.40

**Table 5:** ANOVA analysis for obtained models for MP-PS biodegradation by *Bacillus cereus* and *Pseudomonas alcaligenes*.

Bacterium	Applied Model	Statistical Analysis						Influential Model Factors	Influential Parameters
		Model				Coefficients			
		*R* ^2^	*R* ^2^_adj_	*F*	*p*	Coefficient Value	*p*		
*Bacillus cereus*	MODEL I	0.8616	0.8435	47.71	0.000	*a*_0_ = 7.55	0.000		agitation speed
						*a*_1_ = 1.34 × 10^−4^	0.304		
						*a*_2_ = −9.02 × 10^−5^	0.107		
						*a*_3_ = −6.03 × 10^−3^	0.000	*X*_3_	
	MODEL II	0.9108	0.8636	19.29	0.000	*a*_0_ = 8.16	0.000		agitation speed
						*a*_1_ = 1.30 × 10^−3^	0.271		
						*a*_2_ = −1.57 × 10^−5^	0.091		
						*a*_3_ = −0 × 02	0.000	*X*_3_	
						*a*_4_ = 1.87 × 10^−7^	0.550		
						*a*_5_ = −1.02 × 10^−6^	0.732		
						*a*_6_ = −8.38 × 10^−7^	0.504		
						*a*_7_ = −1.38 × 10^−6^	0.198		
						*a*_8_ = −2.23 × 10^−8^	0.905		
						*a*_9_ = 4.25 × 10^−5^	0.020	*X*_3_^2^	
*Pseudomonas alcaligenes*	MODEL I	0.4287	0.3542	5.75	0.004	*a*_0_ = 7.44	0.000		agitation speed and *OD*
						*a*_1_ = 3.87 × 10^−3^	0.011	*X*_1_	
						*a*_2_ = −3.67 × 10^−4^	0.304		
						*a*_3_ = 1.10	0.008	*X*_3_	
	MODEL II	0.9076	0.8587	18.55	0.000	*a*_0_ = 3.49	0.000		agitation speed, MP size and *OD*
						*a*_1_ = 4.98 × 10^−2^	0.000	*X*_1_	
						*a*_2_ = 4.37 × 10^−4^	0.038	*X*_2_	
						*a*_3_ = 6.93	0.000	*X*_3_	
						*a*_4_ = 2.25 × 10^−6^	0.581		
						*a*_5_ = −1.04 × 10^−2^	0.018	*X*_1_· *X*_3_	
						*a*_6_ = 1.60 × 10^−3^	0.127		
						*a*_7_ = −1.46 × 10^−4^	0.000	*X*_1_^2^	
						*a*_8_ = −2.03 × 10^−6^	0.169		
						*a*_9_ = −8.32	0.000	*X*_3_^2^	

**Table 6:** Optimal conditions for MP-PS biodegradation by *Bacillus cereus* and *Pseudomonas alcaligenes*.

	*Bacillus cereus*			*Pseudomonas alcaligenes*		
Factor	*γ*_MP_/mg/L	MP Size/µm	Agitation Speed/rpm	Agitation Speed/rpm	MP Size/µm	*OD*/-
Value	66.20	413.29	100.45	161.08	334.73	0.35

## Data Availability

The data presented in this study are available on request from the corresponding author.
